# Microwave-Assisted Post-Ugi Reactions for the Synthesis of Polycycles

**DOI:** 10.3390/molecules27103105

**Published:** 2022-05-12

**Authors:** Liangliang Song, Lingchao Cai, Erik V. Van der Eycken

**Affiliations:** 1 Jiangsu Co-Innovation Center of Efficient Processing and Utilization of Forest Resources, College of Chemical Engineering, Nanjing Forestry University, Nanjing 210037, China; cailingchao@njfu.edu.cn; 2 Laboratory for Organic & Microwave-Assisted Chemistry (LOMAC), Department of Chemistry, University of Leuven (KU Leuven), Celestijnenlaan 200F, B-3001 Leuven, Belgium; 3 Peoples’ Friendship University of Russia (RUDN University), Miklukho-Maklaya Street 6, 117198 Moscow, Russia

**Keywords:** microwave, post-Ugi, polycycles, copper, palladium

## Abstract

Microwave irradiation and post-Ugi reactions own their respective advantages in comparison with other strategies. The combination of microwave irradiation and post-Ugi reactions shows paramount importance in the construction of polycycles. This minireview outlines the recent developments of microwave-assisted post-Ugi reactions for the synthesis of polycycles. Through transition metal-catalyzed or transition metal-free transformations, diverse polycycles are prepared in an efficient, rapid, and step-economical manner.

## 1. Introduction

Since the emergence of mono-mode reactors, microwave-assisted chemistry has been increasingly utilized in organic synthesis over the years [1,2,3]. For mono-mode reactors, the electromagnetic irradiation is centralized directly via an accurately programmed wave guide onto the reaction tube, which is fixed at a designed distance from the radiation source. Mono-mode reactors could provide designed and accurate microwave irradiation, avoiding undesired and useless waves. As alternative heating source, microwave irradiation has obtained more attention because of high efficiency and reproducibility [1,2,3]. Microwave heating shortens reaction time from hours to minutes or seconds, offering a more rapid method through uniform and efficient heating than conventional heating. This is generally accompanied with significant decrease of energy consumption, promoting the efficiency and yield of the desired products, and reducing the level of side products [4,5,6].

The Ugi four-component reaction (Ugi-4CR) was discovered by Ugi in 1959 and assembles an amine, an acid, an aldehyde or a ketone, and an isocyanide into α-acylaminoamides through a one-pot reaction [7]. The Ugi-4CR has become one of the highly explored reactions for forming multifunctional adducts, due to the mild reaction conditions, wide scope, and high variability [8,9,10]. Notably, the Ugi-4CR gives a chance for a variety of post-transformations by modifying the four components, usually in two operational steps [11,12,13]. The post-Ugi reactions are well suited for the construction of important heterocycles, macrocycles, polymers, and other compounds in drug discovery and natural product synthesis.

**Scheme 1:**
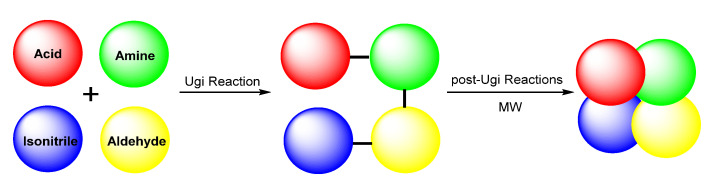
Synthesis of polycycles through microwave-assisted post-Ugi reactions.

As microwave irradiation and post-Ugi reactions possess their respective advantages over other protocols, merging strategies show high value in synthetic chemistry. Through the combination of microwave irradiation and post-Ugi reactions, various polycycles have been efficiently and sustainably synthesized. In this minireview, we wish to highlight the recent advances of microwave-assisted post-Ugi reactions for the synthesis of polycycles (Scheme 1). For the sake of clarity, we have divided this minireview into the following sections: (a) copper catalysis, (b) palladium catalysis, (c) other transition metal catalysis, and (d) transition metal-free catalysis.

## 2. Copper Catalysis

**Scheme 2:**
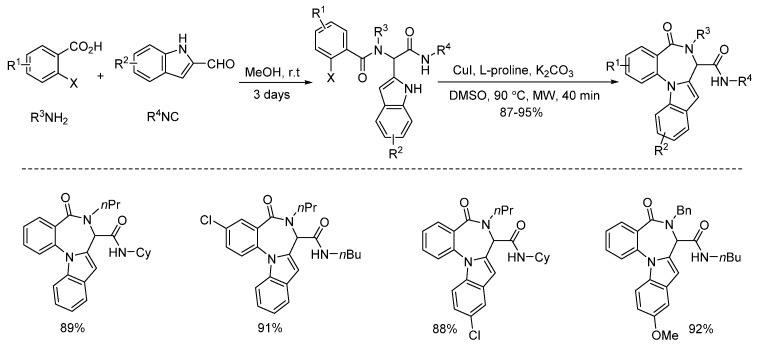
Copper-catalyzed arylation for the synthesis of tetracyclic benzo[*e*][1,4]diazepines.

**Scheme 3:**
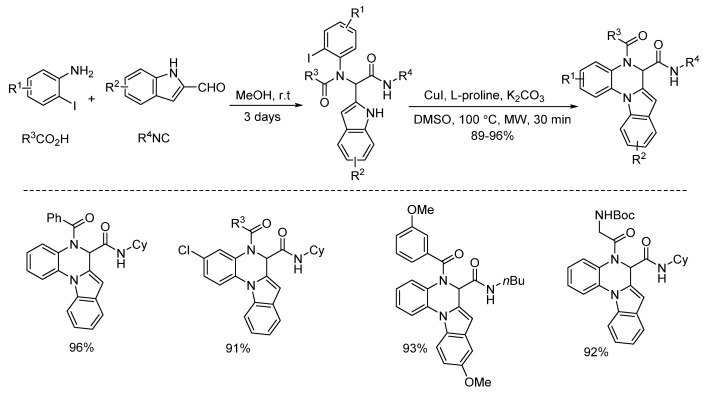
Synthesis of 5,6-dihydroindolo[1,2-a]quinoxalines via copper catalysis.

In 2013, Liu and co-workers developed a copper-catalyzed intramolecular arylation of Ugi-adducts [14]. Under microwave heating, diverse tetracyclic benzo[*e*][1,4]diazepines were synthesized within 40 min in high yields and with excellent chemoselectivities (Scheme 2). Then they changed the amine and acid components, giving a series of new Ugi-adducts [15]. By using the same conditions in 30 min, the Ugi-adducts were performed to deliver various 5,6-dihydroindolo[1,2-*a*]quinoxalines with excellent yields and chemoselectivities (Scheme 3).

**Scheme 4:**
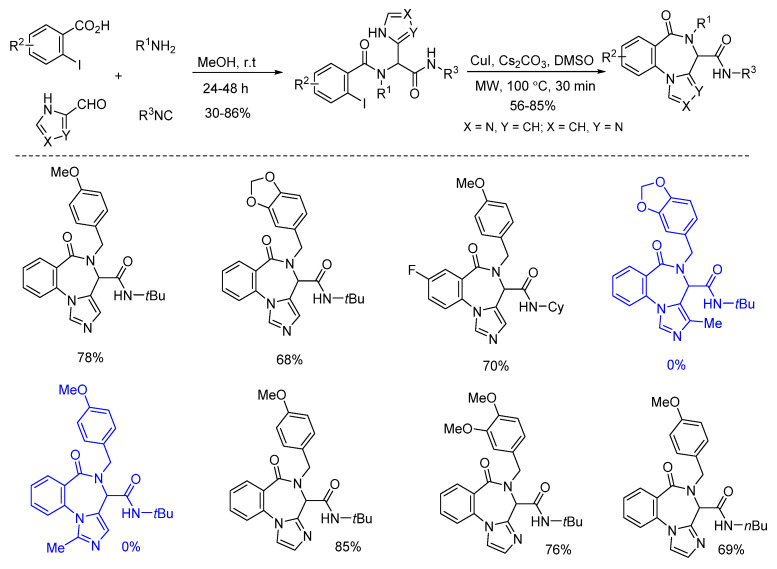
Synthesis of 4*H*-benzo[*f*]imidazo[1,4]diazepin-6-ones through Ullmann coupling.

Van der Eycken’s group reported a post-Ugi copper-catalyzed intramolecular Ullmann coupling in 2014 (Scheme 4). Microwave assistance promoted the diversity-oriented formation of 4*H*-benzo[*f*]imidazo[1,4]diazepin-6-ones with high yields, in 30 min [16]. Ugi-adducts derived from imidazole-4-carbaldehyde and imidazole-2-carbaldehyde reacted smoothly to give the corresponding products. Notably, substrates derived from C-2 or C-5 substituted imidazole-4-carbaldehyde failed to deliver the corresponding products due to the decomposition of the starting materials.

**Scheme 5:**
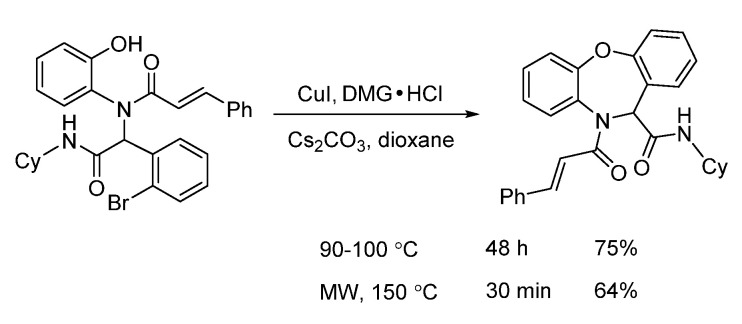
Intramolecular Ullmann etherification under conventional and microwave heating.

**Scheme 6:**
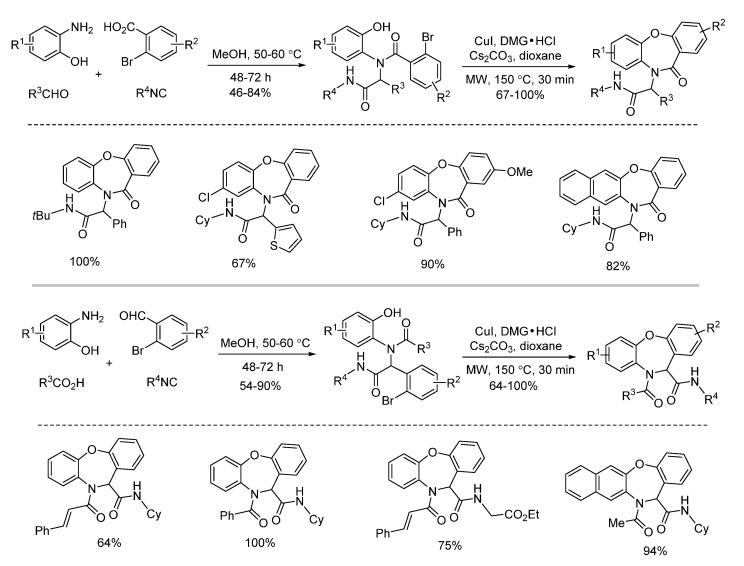
Construction of dibenz[*b,f*][1,4]oxazepines via Ullmann etherification.

In 2016, a microwave-assisted intramolecular Ullmann etherification was established by Dai and co-workers for the efficient construction of dibenz[*b,f*][1,4]oxazepine scaffold. When optimizing the reaction conditions, it was found that conventional heating gave 75% yield after 48 h, while microwave irradiation delivered 64% yield in 30 min, dramatically speeding up the reaction (Scheme 5). Under the optimal reaction conditions of microwave irradiation, they explored the substrate limitation (Scheme 6). By using 2-bromobenzoic acids or 2-bromobenzaldehydes, the diverse 6/7/6-fused tricyclic heterocycles were synthesized from Ugi-adducts via copper-catalyzed Ullmann coupling in 30 min [17]. In contrast with their previous report through copper-catalyzed Goldberg amidation of Ugi-adducts [18], this reaction exhibited excellent chemoselectivity to undergo Ullmann etherification under microwave assistance.

## 3. Palladium Catalysis

**Scheme 7:**
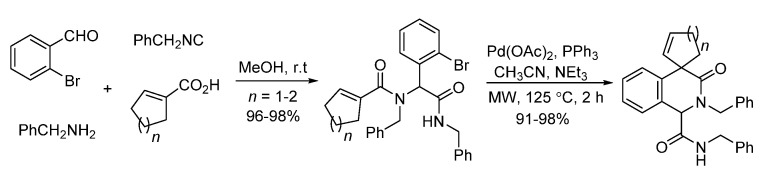
Microwave-assisted intramolecular Heck cyclization of Ugi-adducts.

Gracias and co-workers described a microwave-assisted intramolecular Heck cyclization of Ugi-adducts in 2004 [19]. Through palladium catalysis, highly functionalized N-heterocyclic scaffolds were synthesized with excellent yields in 2 h (Scheme 7).

**Scheme 8:**
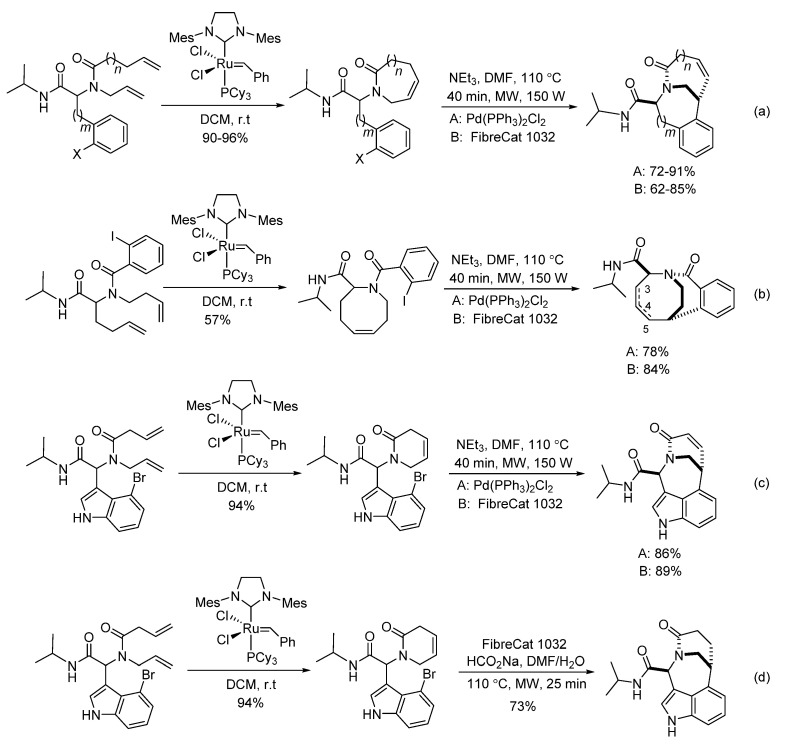
Sequenced RCM/Heck reactions.

In 2007, Judd’s group reported a sequenced RCM/Heck reaction of diverse Ugi-adducts [20]. Under the assistance of microwave heating, various bridged bicyclic lactams were prepared with high yields and diastereoselectivities in 40 min (Scheme 8). Compared to the homogeneous palladium catalyst Pd(Ph_3_P)_2_Cl_2_, the immobilized palladium catalyst FibreCat 1032 showed similar performance for all cases. This method could also give [4.3.2] the bicycloundecane scaffold in high yield and diastereoselectivity, leading to a mixture of the alkene regioisomers (Scheme 8b). The more constrained indole RCM product afforded the bridged indole scaffold with excellent selectivity (Scheme 8c). Interestingly, by using microwave heating, the indole RCM product delivered the saturated bridged bicyclic lactam with high diastereoselectivity using FibreCat 1032 and sodium formate (Scheme 8d).

**Scheme 9:**
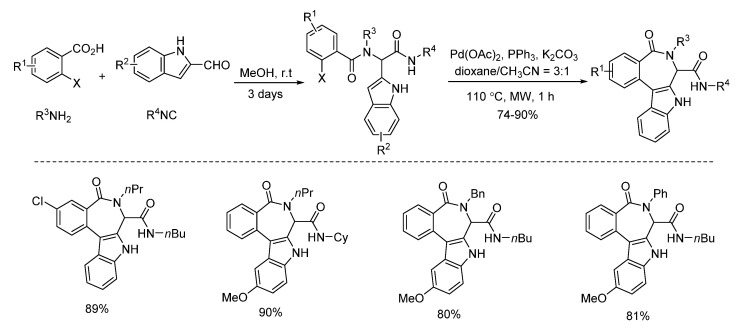
Synthesis of benzo[5,6]azepino[3,4-*b*]indoles through palladium catalysis.

**Scheme 10:**
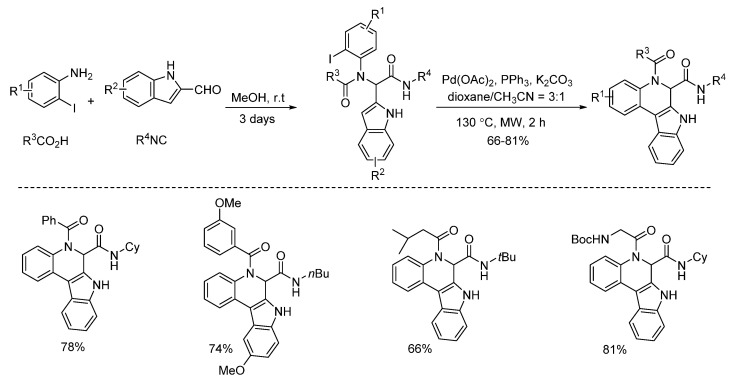
Preparation of indole-fused 6,7-dihydroindolo[2,3-*c*]quinolines.

Under microwave irradiation, palladium and copper catalysts showed distinctly different catalytic properties [14,15]. Highly selective C3-arylation of Ugi-adducts was achieved by Liu’s group in 2013 [14]. Under microwave-assisted palladium catalysis, various benzo[5,6]azepino[3,4-*b*]indoles were constructed with high yields in 1 h (Scheme 9). Subsequently, the same group employed this strategy using the Ugi-adducts derived from 2-halogenated anilines instead of 2-halogenated benzoic acids [15]. With the assistance of microwave irradiation, diverse indole-fused 6,7-dihydroindolo[2,3-*c*]quinolines were obtained with high yields in 2 h (Scheme 10).

## 4. Other Transition Metal Catalysis

**Scheme 11:**
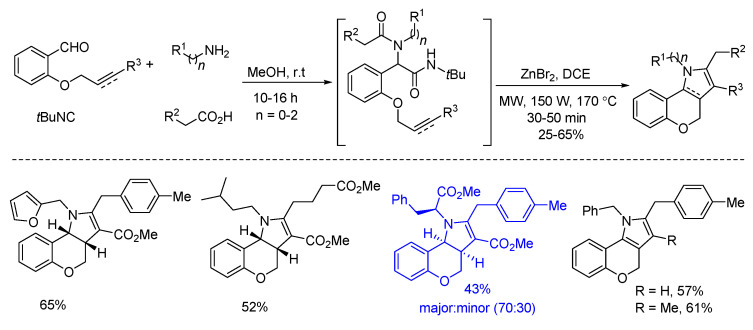
Zinc-catalyzed post-Ugi transformation for the construction of chromenopyrroles.

**Scheme 12:**
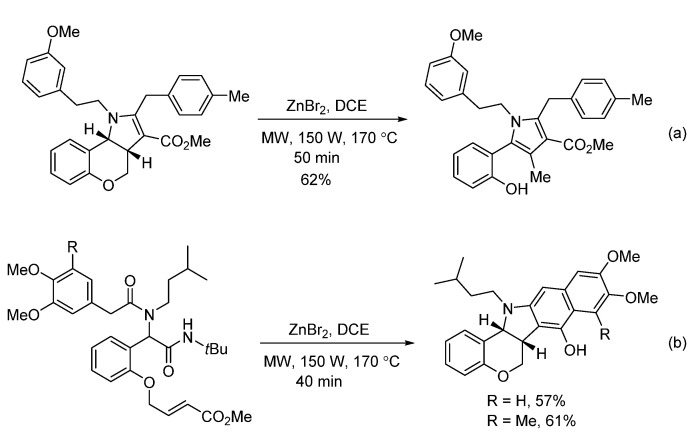
Special examples for zinc-catalyzed transformation.

**Scheme 13:**
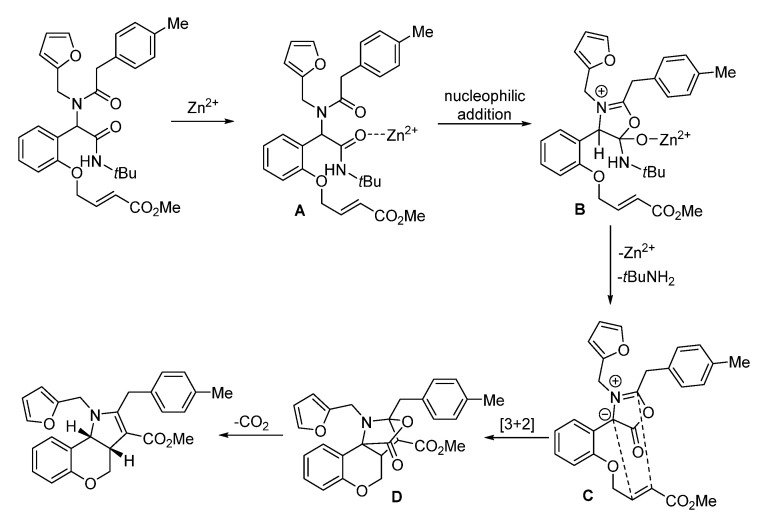
Proposed mechanism for zinc-catalyzed post-Ugi transformation.

In 2018, Sieburth and Al-Tel discovered a zinc-catalyzed microwave-assisted post-Ugi cascade transformation (Scheme 11). Based on a build/couple/pair strategy, the diastereoselective synthesis of various chromenopyrroles was performed in a one-pot fashion in 50 min [21]. By using amino acids as chiral auxiliary, this method provided access to enantiopure chromenopyrroles. The polycyclic product was subjected to similar conditions, resulting in pyrrole formation in 62% yield through C-O bond cleavage and isomerization (Scheme 12a). By using 3,4,5-trimethoxy- and 4,5-dimethoxyphenylacetic acids, pentacyclic products were produced via additional intramolecular Friedel–Crafts acylation (Scheme 12b). As proposed, the Ugi-adduct first coordinates with Zn^2+^, giving intermediate **A** (Scheme 13). This is followed by nucleophilic addition of the tertiary amide and deprotonation, generating the azomethine ylide **C**. Sequential intramolecular [3 + 2] cyclization produces the strained intermediate **D**, which undergoes the expulsion of CO_2_ to give the desired chromenopyrrole product.

**Scheme 14:**
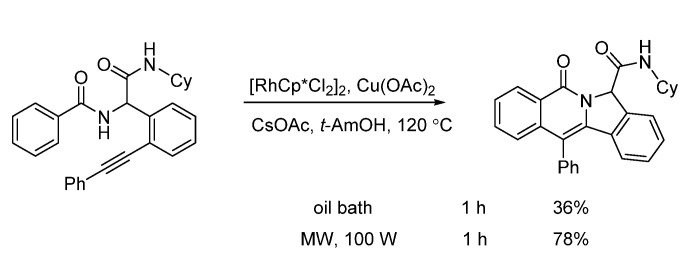
Rhodium-catalyzed annulation under conventional and microwave heating.

**Scheme 15:**
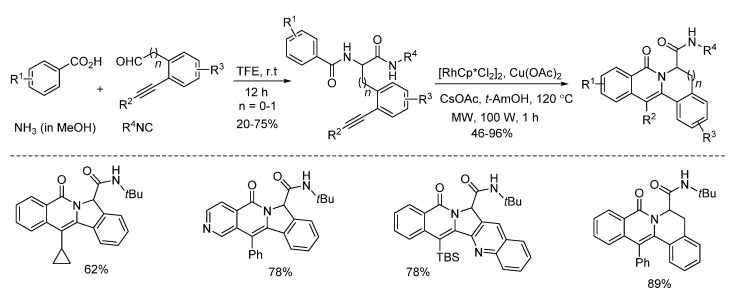
Microwave-assisted rhodium(III)-catalyzed intramolecular annulation.

**Scheme 16:**
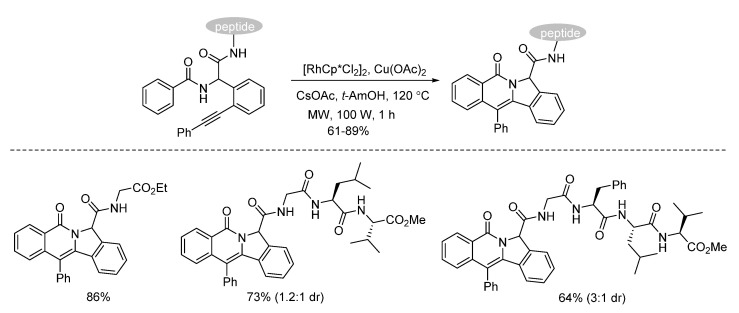
Chemoselective modification of oligopeptides.

Van der Eycken and co-workers developed a microwave-assisted rhodium(III)-catalyzed intramolecular annulation of Ugi-adducts in 2019. It was observed that microwave heating (100 W) afforded the annulation product in 78% yield, while conventional heating only gave 36% yield in the same reaction time (Scheme 14). Through chemoselective C(sp^2^)-H activation (Scheme 15), without installing a directing group, modification of peptidomimetics and oligopeptides was accomplished in a rapid and step-economical fashion, delivering various indolizinone and quinolizinone scaffolds in 1 h (Scheme 16). Furthermore, this method was compatible with water and specifically added N-protected amino acids [22].

**Scheme 17:**
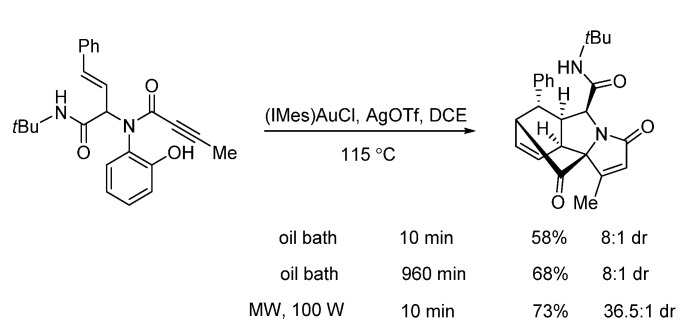
Gold-triggered spirocarbocyclization under conventional and microwave heating.

**Scheme 18:**
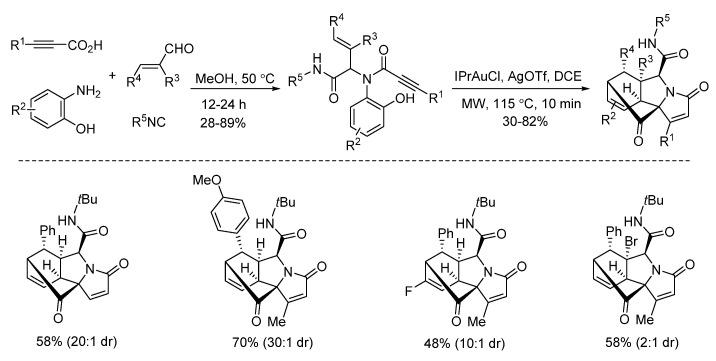
Gold-triggered dearomative spirocarbocyclization/Diels–Alder reaction.

**Scheme 19:**
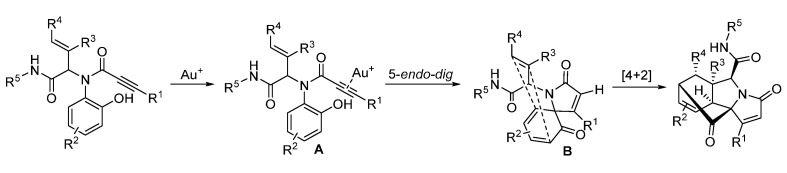
Proposed mechanism for gold-catalyzed post-Ugi transformation.

In 2019, Van der Eycken’s group reported a gold-triggered dearomative spirocarbocyclization/Diels–Alder reaction of Ugi-adducts. When optimizing the reaction conditions, it was observed that microwave irradiation could not only accelerate the reaction and enhance the yield, but also significantly improve the diastereomeric ratio (Scheme 17). By using microwave heating (Scheme 18), the diversity-oriented synthesis of complex bridged polycyclic N-heterocycles was achieved with excellent chemo- and diastereoselectivity, in 10 min [23]. According to the mechanism, the triple bond of the Ugi-adducts is activated by the in situ generated cationic gold(I) species, giving intermediate **A** (Scheme 19). This is followed by dearomatization, generating intermediate **B** in a 5-*endo-dig* fashion. The final products are formed by sequential [4 + 2] intramolecular cycloaddition.

## 5. Transition Metal-Free Catalysis

**Scheme 20:**
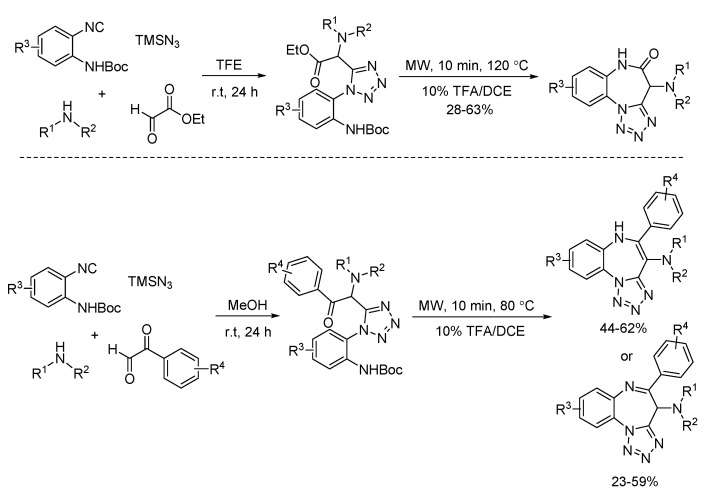
Microwave-assisted deprotection and cyclization process.

In 2012, a simultaneous deprotection and cyclization process of Ugi-azide adducts was developed by Hulme (Scheme 20). By using ethyl glyoxylate or phenylglyoxaldehydes, tricyclic tetrazolo-fused benzodiazepines and benzodiazepinones were prepared [24]. This microwave-assisted transformation could be completed in 10 min.

**Scheme 21:**
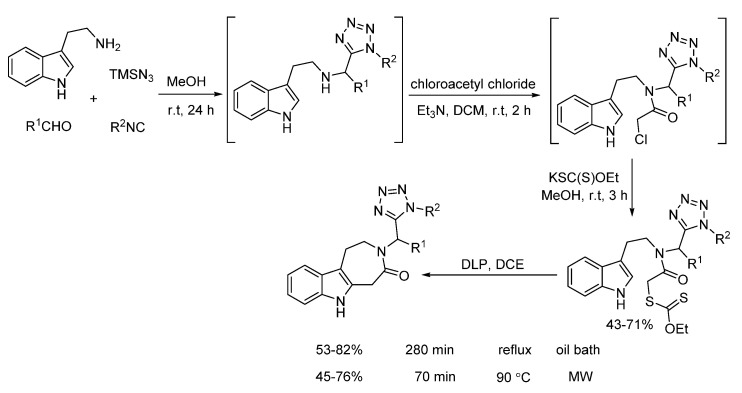
Intramolecular cyclization of xanthates through radical process.

Later on, Gámez-Montaño synthesized the xanthates through one-pot sequential Ugi-azide/N-acylation/S_N_2 reaction (Scheme 21). Under free radical conditions, intramolecular cyclization of xanthates delivered 3-tetrazolylmethyl-azepino[4,5-b]indol4-ones in good yields. Compared to conventional heating requiring 280 min, microwave irradiation could shorten the reaction time to 70 min [25]. Notably, based on a docking study, the final products showed the potential to inhibit 5-Ht_6_ protein.

**Scheme 22:**
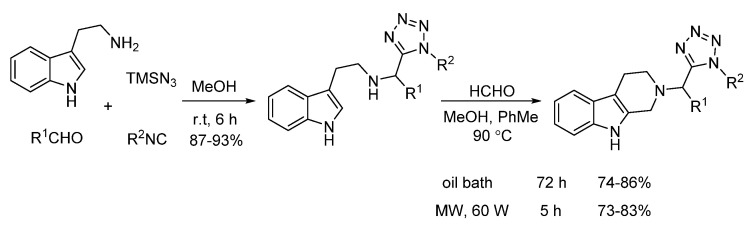
Microwave-assisted Pictet–Spengler reaction of Ugi-azide adducts.

In 2014, El Kaim and Gámez-Montaño reported an efficient Pictet–Spengler reaction of Ugi-azide adducts and formaldehyde (Scheme 22). Under microwave heating, a series of 2-tetrazolylmethyl-2,3,4,9-tetrahydro1H-β-carbolines was synthesized in 73–83% yield in 5 h, while conventional heating gave 74–86% yield in 72 h, indicating that microwave irradiation remarkably shortens the reaction time of this transition metal-free process [26].

**Scheme 23:**
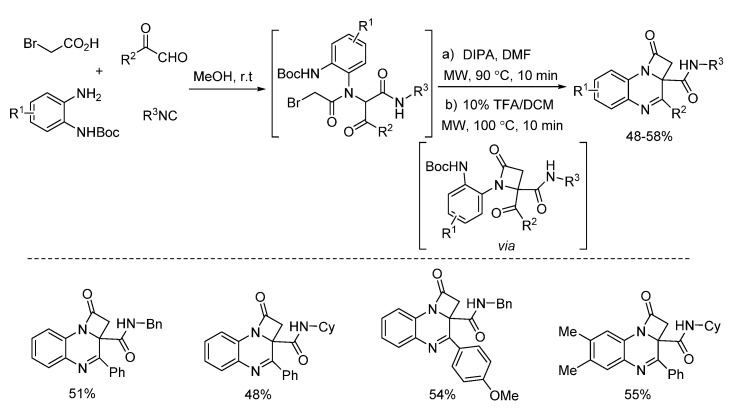
Microwave-assisted synthesis of 2-azetidinones.

**Scheme 24:**
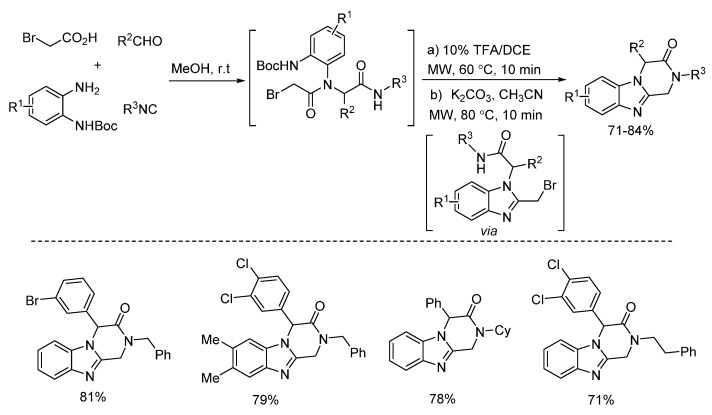
Transition metal-free protocol for the preparation of benzimidazopyrazinones.

In 2018, Zhu’s group presented a one pot post-Ugi cyclization through nucleophilic substitution/deprotection/imine formation cascade (Scheme 23). By using microwave irradiation, different kinds of 2-azetidinones were rapidly synthesized in moderate yields [27]. The 2-azetidinones were screened against diverse cancer cell lines, showing good antitumor activities. Based on this method, the same group reported a similar microwave-assisted post-Ugi cyclization [28]. Through tandem deprotection/nucleophilic addition/nucleophilic substitution process, diverse benzimidazopyrazinones were prepared in a one-pot fashion with high yields (Scheme 24). Under the assistance of microwave heating, this transition metal-free protocol required only one purification procedure after three steps.

## 6. Conclusions

In conclusion, microwave-assisted post-Ugi reactions have been employed for the synthesis of diverse polycycles. From commercially available starting materials, transition metal-catalyzed or transition metal-free transformations are performed in an efficient, rapid, and step-economical manner. With the help of microwave irradiation, linear Ugi-adducts are converted into more complex drug-like polycycles with high chemo-, regio-, and stereoselectivity, exhibiting the robustness and potential of this strategy and unfolding a large window for organic synthesis. We anticipate that this minireview, which focuses on the integration of microwave irradiation and post-Ugi transformations, would provide deep understanding of microwave-assisted post-Ugi reactions and hidden opportunity for the construction of various polycycles.

## Data Availability

Not applicable.
